# Monitoring the Activity of Immobilized Lipase with Quinizarin Diester Fluoro-Chromogenic Probe

**DOI:** 10.3390/molecules22122136

**Published:** 2017-12-04

**Authors:** Carolina Aparecida Sabatini, Denis Massucatto dos Santos, Sabrina Matos de Oliveira da Silva, Marcelo Henrique Gehlen

**Affiliations:** Instituto de Química de São Carlos, Universidade de São Paulo, São Carlos 13566-590, SP, Brazil; carolinasabatini@gmail.com (C.A.S.); denis.massucatto@gmail.com (D.M.d.S.); sabrinacarbureto@gmail.com (S.M.d.O.d.S.)

**Keywords:** fluorochromic substrate, quinizarin diester, lipase assay, enzyme kinetics

## Abstract

Quinizarin diester is used as a fluoro-chromogenic substrate of the activity of lipase supported in poly(methylmetacrylate) beads (CALB, Novozym^®^ 435) dispersed in organic solvents. The monoester and diester of quinizarin are both non-fluorescent species contrasting with the enzymatic product quinizarin that shows optical absorption in the visible region and strong fluorescence signal. The enzymatic conversion is accomplished by spectroscopic measurements and it follows a sigmoid curve from which the mean reaction time of the enzymatic process can be determined. This parameter indicates the enzyme activity of the immobilized lipase. Its dependency with the amount of lipase allowed the determination of the ratio of the catalytic rate and the Michaelis constant (*k_c_*/*K_m_*) and the experimental value found was (1.0 ± 0.1) × 10^−2^ mg^−1^/min in the case of quinizarin diacetate.

## 1. Introduction

The study of lipase catalyzed reactions of hydrolysis or formation of ester is an important issue in organic biosynthesis [1,2,3,4,5]. Moreover, biofuel production from trans-esterification of natural oils and production of enantiomeric compounds using enzymes are applications with great industrial and pharmaceutical interest [6,7,8,9,10,11]. Recent reports in the literature have shown that enzyme immobilization strategies may control the efficiency of the biocatalyst toward improved properties when compared with the behavior of non-immobilized enzyme [12,13,14,15,16].

The enzyme activity may be determined from diverse analytical methods. Among those experimental approaches to monitor enzyme activity, the use of fluorochromic compounds is one of the most practical methods [17,18,19,20,21]. Fluorochromic compounds have established new tools and opened important frontiers in fluorescence spectroscopy and microscopy of enzymes [22,23,24]. The enzyme screening from microorganism is highly effective with the application of fluorescence measurements [25,26,27].

On the other hand, when an immobilized enzyme is reused in a bioreactor, it is important to determine the degree of activity loss in order to decide the replacement of the biocatalyst. Thus, critical and direct methods of enzyme activity evaluation using fluorochromic substrates have become more appealing.

In this contribution, the supported lipase in a catalyzed hydrolysis of aromatic diester is investigated by absorption and emission spectroscopy. We will demonstrate that quinizarin diester can be quantitatively converted to free fluorescent quinizarin (Qz) in the presence of immobilized lipase. This fluorochromic probe allows easy absorption and emission readout in the visible spectral region where scattering and bio-sample damage are less effective contrasting with coumarin based substrates that requires UV excitation.

## 2. Experimental

The preparation of the substrates quinizarin diacetate (QDA) and quinizarin dibutyrate (QDB) was described previously [28]. The purity of the diesters was checked by the absence of the ^1^H-RMN signal at 12.9 ppm of the –OH group (data reported in the Supplementary Materials, SI) of quinizarin and monoester. Also, the UV-visible absorption and fluorescence emission measurements help in the characterization of the compounds.

The supported lipase in poly(methylmetacrylate) beads (CALB, Novozym^®^ 435) was gently provided by Novozymes Co. and it was used as received. The percentage of enzyme in the solid support was calculated from the amount of N found in CHN standard analysis. The experimental samples in fluorescence measurements were prepared by adding 3 mL of the quinizarin diester solution (2.0 × 10^−5^ mol·L^−1^ in hexane, cyclohexane, or in decaline) to a quartz cuvette containing a small amount of immobilized lipase (typically 20 mg of the solid support containing immobilized lipase).

Absorption measurements were performed on a Cary 5G—Varian spectrophotometer, and the corrected steady-state fluorescence spectra were recorded on a CD-900 Edinburgh spectrofluorimeter with excitation at 480 nm. Samples were conditioned in a 1 × 1 cm quartz cuvette and thermostated at 298 K by circulating water through a jacketed cuvette-holder and were keep under constant magnetic stirring. During spectroscopic measurement, the solid beads remained in the bottom of the cuvette and the optical light beam passed though the clear solution region. The emission intensities were calculated from integration of the full emission spectra. Quinizarin diester in solution without addition of the lipase did not undergo detectable hydrolysis during several hours. Fluorescence decays of quinizarin in solution were measured by single-photon timing technique using the picosecond spectrometer described elsewhere [29].

## 3. Results and Discussion

**Figure 1:**
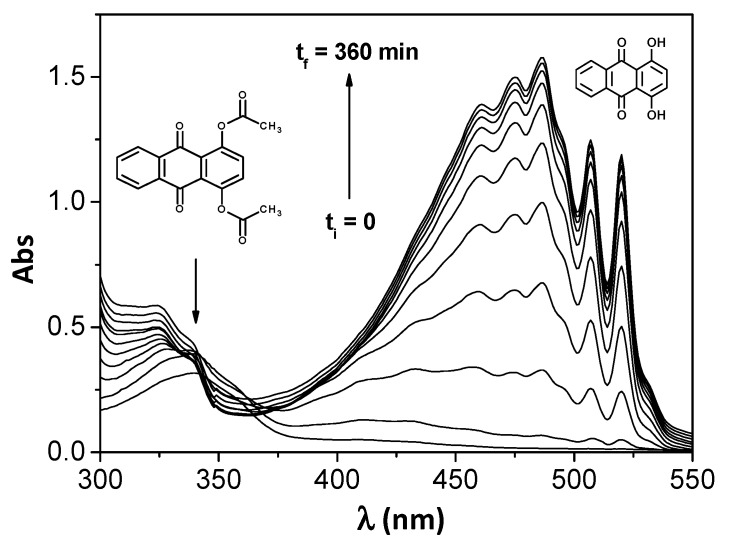
Absorption spectra of quinizarin diacetate (QDA 1.5 × 10^−4^ mol·L^−1^) conversion to quinizarin (Qz) in water saturated cyclohexane containing 20 mg of Novozym 435, T = 298 K.

**Figure 2:**
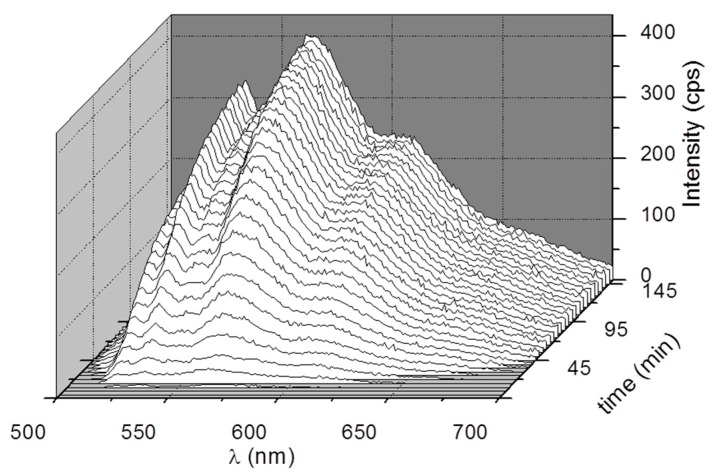
Emission spectra obtained from lipase hydrolysis of QDA 2.0 × 10^−5^ mol·L^−1^ in hexane as a function of reaction time (λ_exc_ = 484 nm, 20 mg of Novozym 435, T = 298 K).

**Figure 3:**
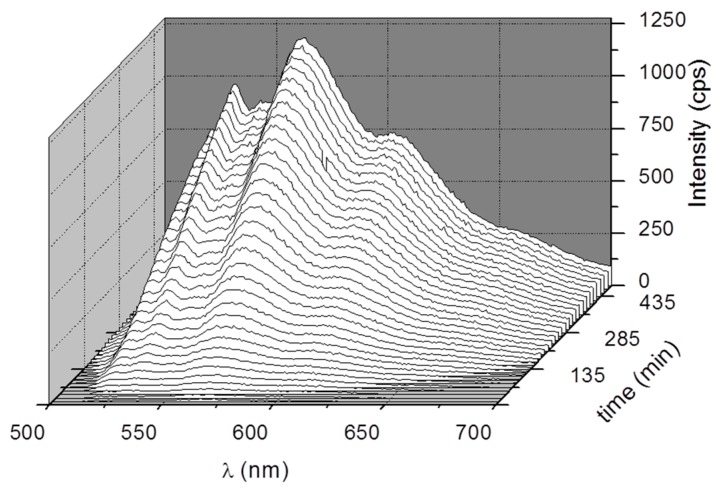
Emission spectra obtained from lipase hydrolysis of QDB 2.0 × 10^−5^ mol·L^−1^ in hexane as a function of reaction time (λ_exc_ = 484 nm, 20 mg of Novozym 435, T = 298 K).

The absorption spectrum of the quinizarin diester (QDA or QDB) solutions in the presence of immobilized lipase in poly(methylmetacrylate) beads (CALB, Novozym^®^ 435) changes dramatically with reaction time as shown in Figure 1 for QDA. The fade of the UV band with maximum at about 330 nm, assigned to the diester species, and the concomitant rise of a strong visible absorption band with maximum about 485 nm of the free quinizarin, points out clearly the lipase activity. The diester and monoester substrates are non-fluorescent species, and only quinizarin is fluorescent. The emission spectrum of the samples with QDA or QDB increases with reaction time generating structured emission band with maximum at 570 nm and a shoulder at about 610 nm as shown in the 3D plot in Figure 2 and Figure 3.

Although the kinetics could be follow by the change in optical density with time, the fluorescence signal is more sensible to the initial conversion and it has no effect of the presence of the intermediate monoester that could introduce some spectral overlap in absorption measurements.

The results in absorption and emission obtained here by enzymatic hydrolysis of quinizarin diesters are similar to the acid hydrolysis used by Ahn et al. to form quinizarin from *tert*-butyloxycarbonyloxy protected dye [30,31].

**Figure 4:**
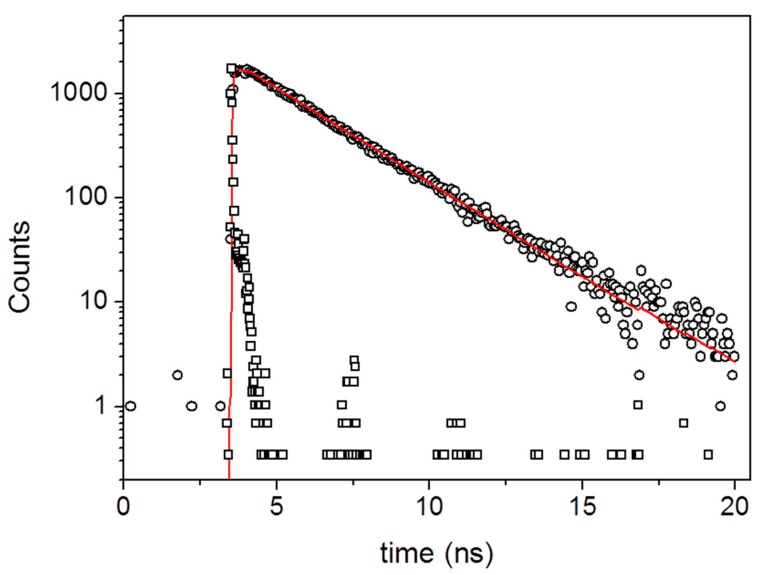
Fluorescence decay obtained from sample under lipase hydrolysis of QDA 2.0 × 10^−5^ mol·L^−1^ in hexane (λ_exc_ = 400 nm, λ_em_ = 550 nm, 20 mg of Novozym 435, T = 298 K). Fluorescence lifetime of 2.3 ns and fitting chi-square χ^2^ = 1.041.

Moreover, the lifetime of the solutions under reaction is about 2.3 ns (monoexponential decay as illustrated in Figure 4) confirming the absence of emission from quinizarin diester or from its intermediate monoester. This lifetime value agrees with the lifetime of pure quinizarin of 2.4 ns in cyclohexane reported in literature [32,33].

**Figure 5:**
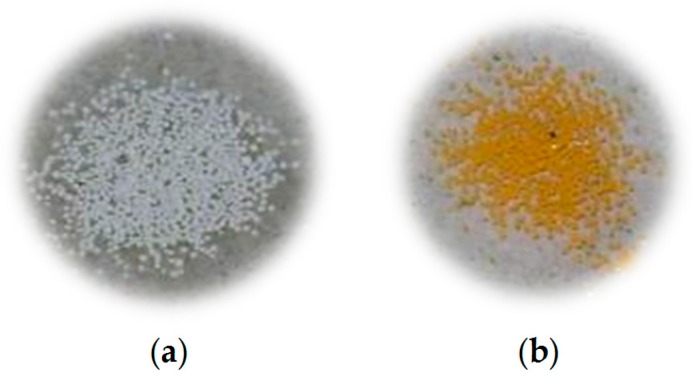
Supported lipase in poly(methylmetacrylate) beads (CALB, Novozym^®^ 435) before (**a**) and after (**b**) quinizarin diester (QDA) addition, reaction and solvent evaporation.

Thus, the quinizarin diester substrate in apolar solvent (water saturated solvent) upon addition of lipase beads undergoes a hydrolysis reaction indicated by both absorption and emission changes of the samples. The solution of diester substrate is colorless, but it becomes yellow and even light orange when the conversion to quinizarin occurs by lipase action. Such clear change can also be eye detected by evaporating the solvent after reaction producing orange polymer beads as illustrated by Figure 5.

**Figure 6:**
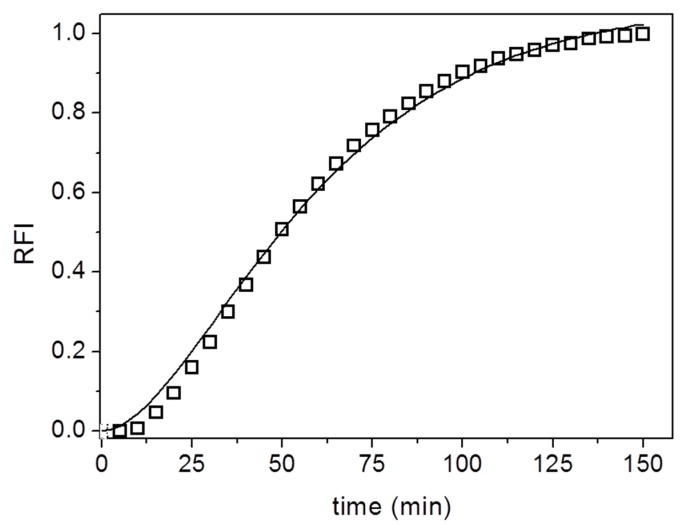
Relative fluorescence intensity (RFI) from integrated emission spectra obtained from lipase hydrolysis of QDA 2.0 × 10^−5^ mol·L^−1^ in hexane as a function of reaction time (λ_exc_ = 484 nm, 20 mg of Novozym 435). T = 298 K. The line is the fitting with the model given by Equation (1) (fitting quality evaluated by R^2^ > 0.98).

**Figure 7:**
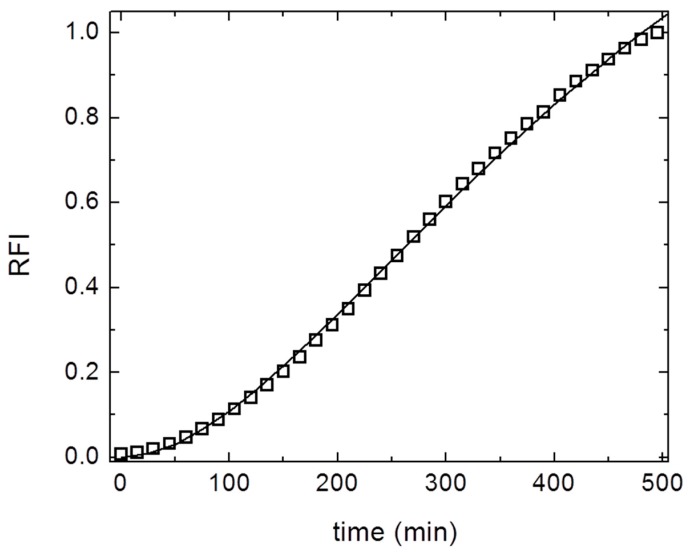
Relative fluorescence intensity (RFI) from integrated emission spectra obtained from lipase hydrolysis of QDB 2.0 × 10^−5^ mol·L^−1^ in hexane as a function of reaction time (λ_exc_ = 484 nm, 20 mg of Novozym 435). T = 298 K. The line is the fitting with the model given by Equation (1) (fitting quality evaluated by R^2^ > 0.98).

When spectral emission of the data in Figure 2 and Figure 3 are integrated and plotted against time, the curve shapes of the relative intensity are not that of a saturation-type growth function as would be expected, but a sigmoid behavior is observed. Typical change of the integrated fluorescence intensity with reaction time is given in Figure 6 in the case of hydrolysis of QDA in hexane, and in Figure 7 in the case of hydrolysis of QDB. In addition, the same sigmoid shape is also observed in the plot of absorbance at 485 nm as a function of time using data in Figure 1 (see Supplementary Materials Figure S3). Thus, the conversion of the diester to the fluorescent quinizarin goes though the intermediate monoester. The conversion of QDB to quinizarin (Qz) is slower when compared with the hydrolysis rate of QDA under similar conditions.

**Figure 8:**
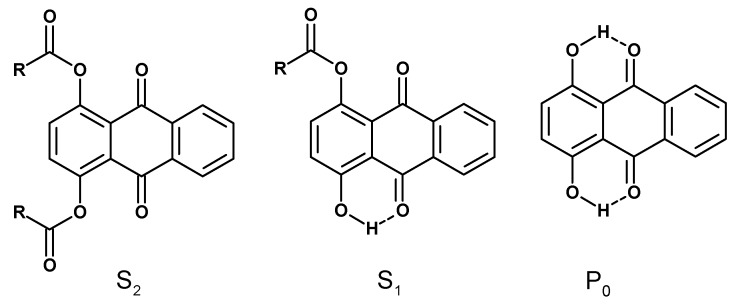
Molecular structures of the diester (S_2_) monoester (S_1_) and quinizarin product (P_0_).

The structures of species in solution during hydrolysis, the diester (S_2_), monoester (S_1_) and quinizarin (P_0_), are given in scheme of Figure 8.

**Scheme 1:**
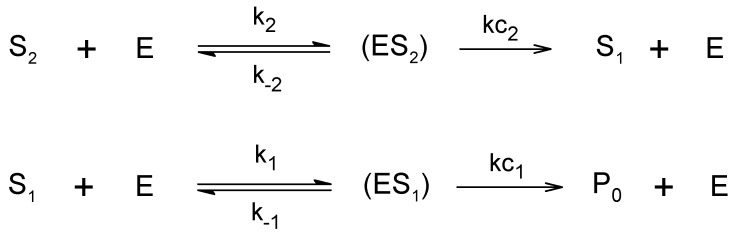
Enzymatic reaction mechanism with two sequential Michaelis-Menten steps.

The S_2_ and S_1_ substrates are non-fluorescent species because their excited state of low energy have a strong nπ* character, while quinizarin has emission in apolar solvents from a low energy ππ* singlet state due to the increase the aromatic resonance of the dye provided by the two intramolecular H-bonds formed (see P_0_ structure in Figure 8) [32,33]. Thus, the fluorescence intensity can grow from a sequential hydrolysis of the quinizarin diester and monoester intermediate, a fact that may explain the S shape observed. A kinetic mechanism where two sequential Michaelis-Menten kinetics are arranged to described the double hydrolysis of the diester is represented in the reaction Scheme 1 below.

In the proposed model, E is the lipase, and ES_2_ and ES_1_ are the enzyme substrate complexes with diester and monoester, respectively. k_1,2_ and k_−1,−2_ represent the association and dissociation rate constant of the complexes ES_1,2_. The catalytic rate constants of first and second hydrolysis step are represented by k_c1,2_. For symmetrical diester as in the present case, the rate constants of association, dissociation, and enzymatic catalysis of the double hydrolysis mechanism may be similar in the first and in the second steps. Thus under such assumption, the kinetics becomes a function of a single parameter *k* = *k_c_*[*E*]/*K_m_*, where k_c_ is the general catalytic rate constant, [*E*] is the enzyme concentration and *K_m_* is the Michaelis-Menten parameter. In this situation, an approximate solution of the fluorescence intensity may be written as,

(1) $$I(t)=I_{\infty }\left(1-(1+k^{*}t)\exp[-k^{*}t]\right)$$

where *I*_∞_ is the fluorescence intensity in the saturation (long time limit) or the maximum intensity observed, and k* is a first order rate constant that scales with *k*. In the present model, the hydrolysis of the diester in the experimental condition used is considered non reversible due to the stabilization of quinizarin by the intramolecular hydrogen bonding in apolar solvent (see Figure 8).

The experimental results of hydrolysis of quinizarin diacetate and dibutyrate in hexane solution are illustrated in Figure 6 and Figure 7 with data fitting using Equation (1), respectively. We observe a good performance of the proposed model to describe the kinetics of the lipase hydrolysis of the diester. The global rate k* of these two systems in hexane is calculated as 2.8 × 10^−2^ min^−1^ and 4.7 × 10^−3^ min^−1^, for QDA and QDB, respectively. Considering that the average reaction time may be defined as 2/k*, (see Supplementary Materials) the corresponding values obtained experimentally are about 71 min and 426 min for QDA and QDB substrates in the presence of 20 mg of Novozym 435 supported lipase, respectively.

A simple analysis of Equation (1) reveals an interesting behavior for the initial rate of the enzymatic process. The expansion of the exponential term of Equation (1) results in a quadratic dependence the relative fluorescence intensity with initial reaction time and enzyme concentration [*E*] (assuming that k* is proportional to *k*).

(2) $$\frac{I(t)}{I_{\infty }}\approx {\left(k_{c}[E]t/K_{m}\right)}^{2}$$

**Figure 9:**
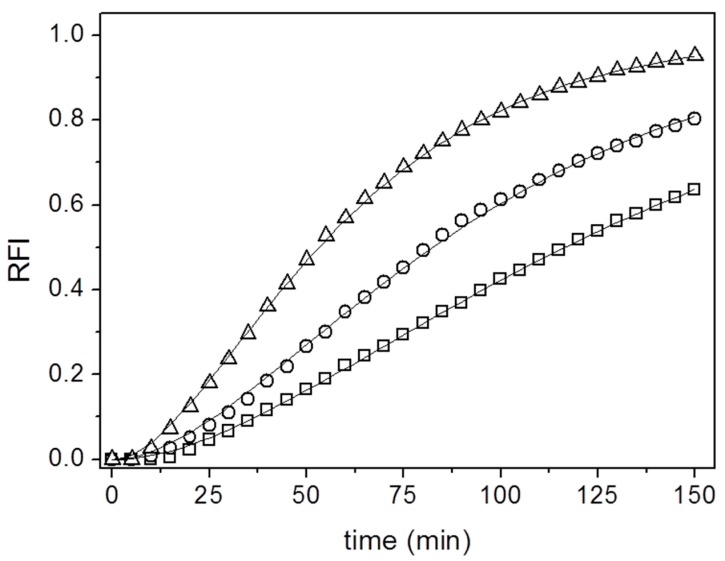
Relative fluorescence intensity obtained from lipase hydrolysis of QDA 2.0 × 10^−5^ mol·L^−1^ in 3 mL of decaline as a function of reaction time and amount of supported lipase added converted to enzyme mass (□) 0.7 mg, (○) 1.5 mg, (Δ) 2.2 mg. The line curves are the fitting of the data with Equation (1). λ_exc_ = 484 nm (fitting quality evaluated by R^2^ was always >0.98).

Experiments of hydrolysis of QDA performed with different amount of supported enzyme in decalin are illustrated in Figure 9. All of these results follow quadratic dependence of initial relative fluorescence intensity (RFI) with reaction time, and they are well fitted with the proposed model of Equation (1) (see curve fitting in Figure 9).

**Figure 10:**
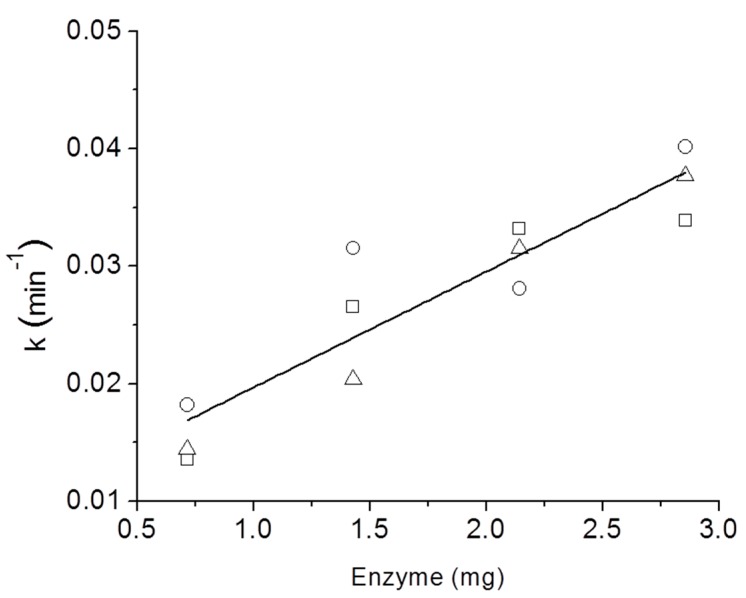
First order rate constant k* of the enzymatic hydrolysis of QDA by lipase as a function of the amount of supported lipase added (Novozym 435) converted to enzyme mass in three apolar solvents. cyclohexane (○), decalin (Δ) and hexane (□). Solution volume of 3 mL, T = 298 K.

The change of k* with enzyme amount for QDA hydrolysis is plotted in Figure 10 for different solvent used. The empirical rate constant k* increases with the amount of added enzyme, with a similar behavior in the three nonpolar solvents. The lipase amount in the supported matrix was determined as 7.2% in weight. Such result was calculated by the CHN analysis of 1.2% of N in the solid samples and using the amino-acid composition of lipase with molecular weight of 33 kDa previously reported [34]. Using this information, it was possible to estimate the amount of enzyme in mg used in the experiments with different mass of supported lipase (Novozym^®^ 435).

Considering that substrates and product are in low concentration and are highly soluble in the organic phase, there will be no saturation of the enzyme capacity in the condition used in our experiments, and the concentration of enzyme non-complexed with the substrate will be slightly small but proportional to total enzyme concentration. It follows that the slope of the plot of Figure 8 is an estimation of *k_c_*/*K_m_* and the value found is (1.0 ± 0.1) × 10^−2^ mg^−1^/min. This rate is one hundred times slower than the hydrolysis rate of *p*-nitrophenyl butyrate by CALB Novozym^®^ 435, a standard UV chromogenic substrate commonly used in the activity test of lipase [35]. It means that lipase turnover in a double hydrolysis of a larger aromatic diester as in the case of quinizarin is a slow process when compared with hydrolysis rate of *p*-nitrophenyl butyrate. On the other hand, lipase immobilization may result in less enzyme activity in ester hydrolysis but on the other hand, it usually gives more enzyme stability and possibility of reuse of the biocatalyst [9]. Further investigation of our system by fluorescence optical microscopy of in situ lipase beads could provide a much faster and reliable method of direct enzyme activity determination in the visible region using quinizarin diester as fluorochromogenic probe.

## 4. Conclusions

The enzymatic hydrolysis of quinizarin diester by supported lipase beads (CALB Novozym^®^ 435) producing the highly fluorescent quinizarin was studied by measuring the relative fluorescence intensity with reaction time in the cases of methyl and butyl derivatives. We showed that quinizarin diesters are potential fluorochromic probes to investigate the activity of immobilized lipase or to check whether the enzyme beads are still active to be reused in organic biosynthesis. The fitting of the fluorescence intensity using a functional with a single parameter k* gave satisfactory representation of the time evolution of quinizarin formation and allowed the estimation of the average reaction time of the immobilized lipase in the biocatalytic process.

## Supplementary Materials

Supplementary materials are available online. Supplementary data concerning ^1^H-NMR spectra of the compounds, and the numerical solution of the set of reaction rates of the enzymatic mechanism expressed in Scheme 1 and comparison with Equation (1) are available free of charge as PDF file.

## References

[1] Villeneuve P., Muderhwa J.M., Graille J., Haas M.J. (2000). Customizing lipases for biocatalysis: A survey of chemical, physical and molecular biological approaches. J. Mol. Catal. B Enzym..

[2] Hoffmann I., Silva V.D., Nascimento M.D. (2011). Enantioselective resolution of (*R*,*S*)-1-phenylethanol catalyzed by lipases immobilized in starch films. J. Braz. Chem. Soc..

[3] Lima G.V., Silva M.R., Fonseca T.S., Lima L.B., Oliveira M.C.F., Lemos T.L.G., Zampieri D., Santos J.C.S., Rios N.S., Gonçalves L.R.B. (2017). Chemoenzymatic synthesis of (*S*)-Pindolol using lipases. Appl. Catal. A.

[4] Khelassi M.M., Zaidi A., Zouioueche L.A. (2017). CAL-B-Catalyzed deacylation of benzylic acetates: Effect of amines addition. Comparison of several approaches. Enzym. Microbial. Technol..

[5] Magadum D.B., Yadav G.D. (2017). Enantioselective resolution of (*R*,*S*)-α-methyl-4-pyridinemethanolusing immobilized biocatalyst: Optimization and kinetic modeling. Biochem. Eng. J..

[6] Al-Suhair S. (2005). Production of biodiesel by lipase-catalyzed transesterification of vegetable oils: A kinetics study. Biotechnol. Prog..

[7] Gamba M., Lapis A.A.M., Dupont J. (2008). Supported ionic liquid enzymatic catalysis for production of biodiesel. Adv. Synth. Catal..

[8] Contesini F.J., Lopes D.B., Macedo G.A., Nascimento M.D., Carvalho P.D. (2010). *Aspergillus* sp. Lipase: Potential biocatalyst for industrial use. J. Mol. Catal. B Enzym..

[9] Verma M., Puri M., Barrow C. (2016). Recent trends in nanomaterials immobilised enzymes for biofuel production. Crit. Rev. Biotechnol..

[10] Lu J., Jin Q., Wang X., Wang X. (2017). Preparation of medium and long chain triacylglycerols by lipase-catalyzed interesterification in a solvent-free system. Process Biochem..

[11] Pascacio V.G.T., Ortíz J.J.V., Pérez M.J., Yates M., Sanchez B.T., Quintero A.R., Lafuente R.F. (2017). Evaluation of different lipase biocatalysts in the production of biodiesel from used cooking oil: Critical role of the immobilization support. Fuel.

[12] Schöffer J.N., Matte C.R., Charqueiro D.S., Menezes E.W., Costa T.M.H., Benvenutti E.V., Rodrigues R.C., Hertz P.F. (2017). Directed immobilization of CGTase: The effect of the enzyme orientation on the enzyme activity and its use in packed-bed reactor for continuous production of cyclodextrins. Process Biochem..

[13] Su F., Li G.L., Fan Y.L., Yan Y.J. (2015). Enhancing biodiesel production via a synergic effect between immobilized *Rhizopus oryzae* lipase and Novozym 435. Fuel Process. Technol..

[14] Santos J.C.S., Rueda N., Gonçalves L.R.B., Lafuente R.F. (2015). Tuning the catalytic properties of lipases immobilized on divinylsulfone activated agarose by altering its nanoenvironment. Enzym. Microb. Technol..

[15] Rodrigues J., Canet A., Rivera I., Osório N.M., Sandoval G., Valero F., Dias S.F. (2016). Biodiesel production from crude Jatropha oil catalyzed by non-commercial immobilized heterologous *Rhizopus oryzae* and *Carica papaya* lipases. Bioresour. Technol..

[16] Rios N.S., Pinheiro M.P., Santos J.C.S., Fonseca T.S., Lima L.D., Mattos M.C., Freire D.M.G., Júnior I.J.S., Aguado E.R., Gonçalves L.R.B. (2016). Strategies of covalent immobilization of a recombinant Candidaantarctica lipase B on pore-expanded SBA-15 and its application in the kinetic resolution of (*R*,*S*)-Phenylethyl acetate. J. Mol. Catal. B Enzym..

[17] Haugland R.P., Johnson I.D. (1993). Detecting enzymes in living cells using fluorogenic substrates. J. Fluoresc..

[18] Lauria S., Casati S., Ciuffreda P. (2015). Synthesis and characterization of a new fluorogenic substrate for nonoacylglycerol lipase and application to inihibition studies. Anal. Bioanal. Chem..

[19] Hendrickson H.S. (1994). Fluorescence-based assays of lipases, phospholipases, and other lipolytic enzymes. Anal. Biochem..

[20] Tallman K.R., Beatty K.E. (2015). Far-red fluorogenic probes for esterase and lipase detection. ChemBioChem.

[21] Prim N., Sánchez M., Ruiz C., Javier Pastor F.I., Diaz P. (2003). Use of methylumbeliferyl-derivative substrates for lipase activity characterization. J. Mol. Catal. B Enzym..

[22] Flomenbom O., Velonia K., Loss D., Masuo S., Cotlet M., Engelborghs Y., Hofkens J., Rowan A.E., Nolte R.J.M., van der Auweraer M. (2005). Stretched exponential decay and correlations in the catalystic activity of fluctuating single lipase molecules. Proc. Nat. Acad. Sci. USA.

[23] Liebherr R.B., Gorris H.H. (2014). Enzyme molecules in solitary confinement. Molecules.

[24] Ursoiu A., Paul C., Kurtán T., Péter F. (2012). Sol-gel entrapped candida antarctica lipase B—A biocatalyst with excelente stability for kinetic resolution of secondary alcohols. Molecules.

[25] Yang Y., Babiak P., Reymond J.-L. (2006). New nonofunctionalized fluorescein derivatives for the efficient high-throughput screening of lipases and esterases in aqueous media. Helv. Chim. Acta.

[26] Mantovani S.M., de Oliveira L.G., Marsaioli A.J. (2008). Whole cell quick E for epoxide hydrolases screening using fluorescente probes. J. Mol. Catal. B Enzym..

[27] Mantovani S.M., de Oliveira L.G., Marsaioli A.J. (2010). Esterase screening using whole cells of brazilian soil microorganisms. J. Braz. Chem. Soc..

[28] Sabatini C.A., Gehlen M.H. (2014). Enzymatic hydrolysis of quinizarin diester by lipase in silica nanoparticles investigated by fluorescence microscopy. J. Nanopart. Res..

[29] Pereira R.V., Gehlen M.H. (2006). Photoinduced intramolecular charge transfer in 9-aminoacridinium derivatives assisted by intramolecular H-bond. J. Phys. Chem. A.

[30] Kim J.M., Kang J.H., Han D.-K., Lee C.W., Ahn K.D. (1998). A novel precursor for color and fluorescence imaging. Chem. Mater..

[31] Ahn K.D., Yoo K.W., Soh J.H., Kang J.H. (2009). Fluorescent photoimaging with polymer having protected quinizarin dye precursors by a dry process based on chemical amplification. React. Funct. Polym..

[32] Pal H., Palit D.K., Mukherjee T., Mittal J.P. (1990). Interaction of the excited singlet state of disubstituted anthraquinones with aromatic hydrocarbons: A fluorescence-quenching study. Chem. Phys. Lett..

[33] Palit D.K., Pal H., Mukherjee T., Mittal J.P. (1990). Photodynamics of the S_1_ state of some hydroxyl-and amino-substituted naphthoquinones and anthraquinones. J. Chem. Soc. Faraday Trans..

[34] Uppenberg J., Hansen M.T., Patkar S., Jones T.A. (1994). The sequence, crystal structure determination and refinement of two crystal forms of lipase B from candida Antarctica. Structure.

[35] Costa L., Brissos V., Lemos F., Ribeiro F.R., Cabral J.M.S. (2008). Comparing the effect of immobilization methods on the activity of lipase biocatalysts in ester hydrolysis. Bioprocess Biosyst. Eng..

