# Supplementary material for: Monitoring the Activity of Immobilized Lipase with Quinizarin Diester Fluoro-Chromogenic Probe
Source: Molecules. 2017 Dec 4;22(12):2136. doi: 10.3390/molecules22122136 (PMC6149872; doi:10.3390/molecules22122136)
Supplement: Supplementary file 1 [file molecules-22-02136-s001.pdf]

Monitoring the activity of immobilized lipase with quinizarin diester fluoro-chromogenic probe

Carolina Aparecida Sabatini, Denis Massucatto dos Santos, Sabrina Matos de Oliveira da Silva, Marcelo Henrique Gehlen*

Instituto de Química de São Carlos, Universidade de São Paulo

13566-590, São Carlos, SP, Brazil

*e-mail: marcelog@iqsc.usp.br

Supplementary Information

Considering the reaction mechanism in scheme 1, the following rate equation system can be written,

$$\frac{d[S_2]}{dt} = k_{-2}[ES_2] - k_2[S_2][E] \qquad \text{(S1)}$$

$$\frac{d[S_1]}{dt} = k_{-1}[ES_1] + k_{c2}[ES_2] - k_1[S_1][E] \qquad \text{(S2)}$$

$$\frac{d[P_0]}{dt} = k_{c1}[ES_1] \qquad \text{(S3)}$$

$$\frac{d[ES_2]}{dt} = -(k_{-2} + k_{c2})[ES_2] + k_2[S_2][E] \qquad \text{(S4)}$$

$$\frac{d[ES_1]}{dt} = -(k_{-1} + k_{c1})[ES_1] + k_1[S_1][E] \qquad \text{(S5)}$$

$$\frac{d[E]}{dt} = (k_{-1} + k_{c1})[ES_1] + (k_{-2} + k_{c2})[ES_2] - (k_1[S_1] + k_2[S_2])[E] \qquad \text{(S6)}$$

The numerical solution of the rate equation system above was obtained using the fourth-order Runge-Kutta integration method. For symmetrical diester, the rate constants of association, dissociation, and enzymatic catalysis of the double hydrolysis mechanism are assumed equal in the first and in the second hydrolytic steps. The normalized concentration of $P_0(t)$ is considered proportional to the relative fluorescence intensity in dilute solution. The simulated profiles of the relative fluorescence intensity and the fitting of the numerical data with empirical function given by equation 1 are illustrated in Figures S1 and S2.

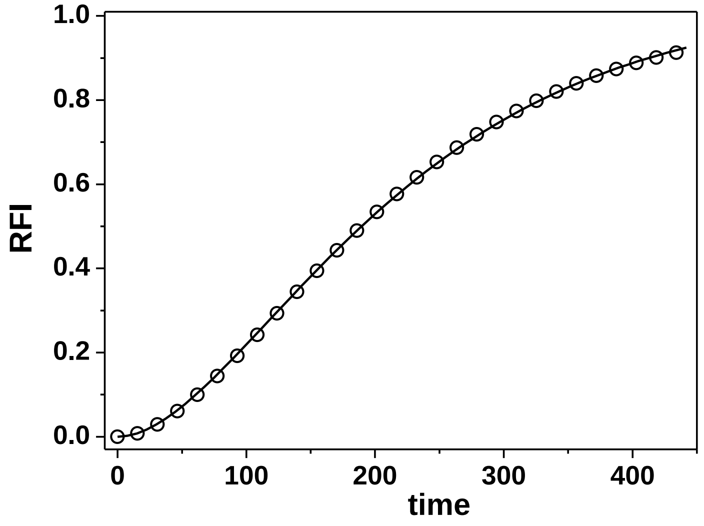

Figure S1. Simulation of the enzyme kinetics and relative fluorescence intensity calculation assuming: $S_0^2 = 1.0$, $E_0 = 0.5$, $k_1 = 0.4$, $k_{-1} = 3.0$, $k_c = 0.1$ and $K_m = 7.7$. Using these parameters values, k = 0.00645. The numerical solution of the rate equation system using fourth-order Runge-Kutta integration method is represented by (○). The continuous line is a non linear fitting of the numerical points using equation 1 providing a fitting parameter k* = 0.00582.

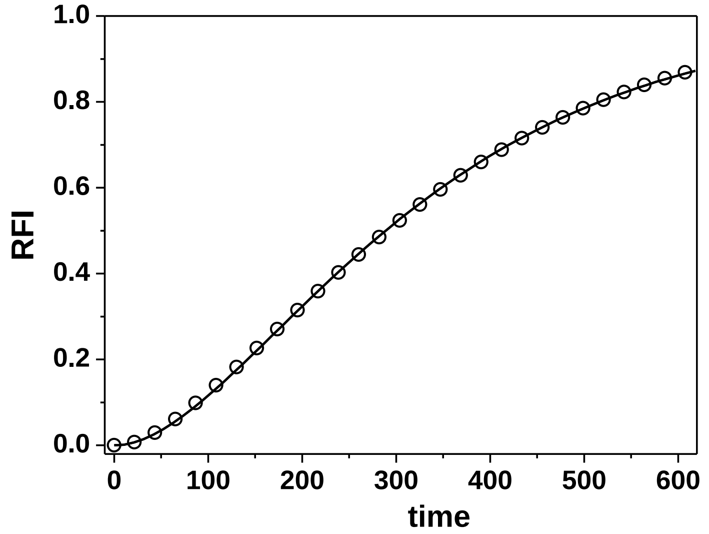

Figure S2. Simulation of the enzyme kinetics and relative fluorescence intensity calculation assuming: $S_0^2 = 1.0$, $E_0 = 0.5$, $k_1 = 0.7$, $k_{-1} = 3.0$, $k_c = 0.1$ and $K_m = 4.4$. Using these values parameter k = 0.0113. The numerical solution of the rate equation system using fourth-order Runge-Kutta integration method is represented by (○). The continuous line is a non linear fitting of the numerical points using equation 1 providing a fitting parameter k* = 0.0086.

Thus, the function given by equation 1 approached quite well to the numerical values in all practical conditions tested (see the continuous line in figures S1 and S2 for instance). This means that a fitting parameter defined as k* which scales with k of the stationary condition may be recovered from analysis of experimental data. Moreover, the average reaction time is the integral over time of the survival fraction of substrates,

$$\tau = \int_0^\infty \left(1 - [P_0]/S_2^0\right)\, dt = \int_0^\infty (1 + k^* t)\exp[-k^* t]\; dt = 2/k^* \qquad \text{(S7)}$$

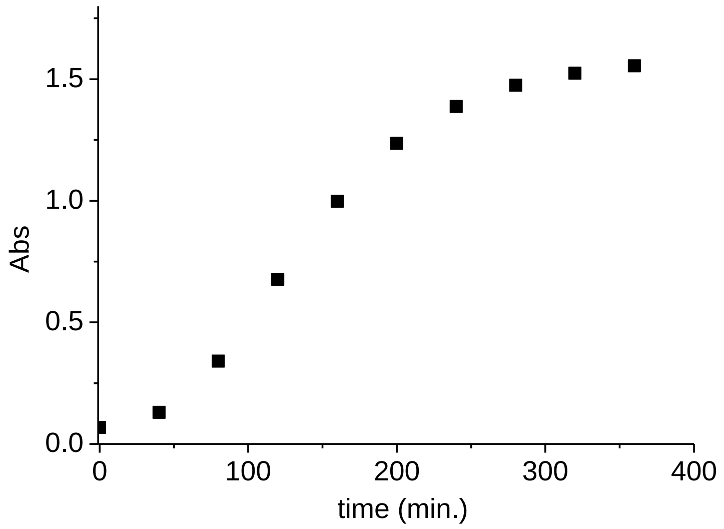

Figure S3. Sigmoidal shape of the of the absorbance curve at 485 nm due to quinizarin formation upon hydrolysis of quinizarin diacetate (QDA $1.5x10^{-4}$ mol L^{-1}) in water saturated cyclohexane containing 20 mg of Novozym 435, T = 298K.

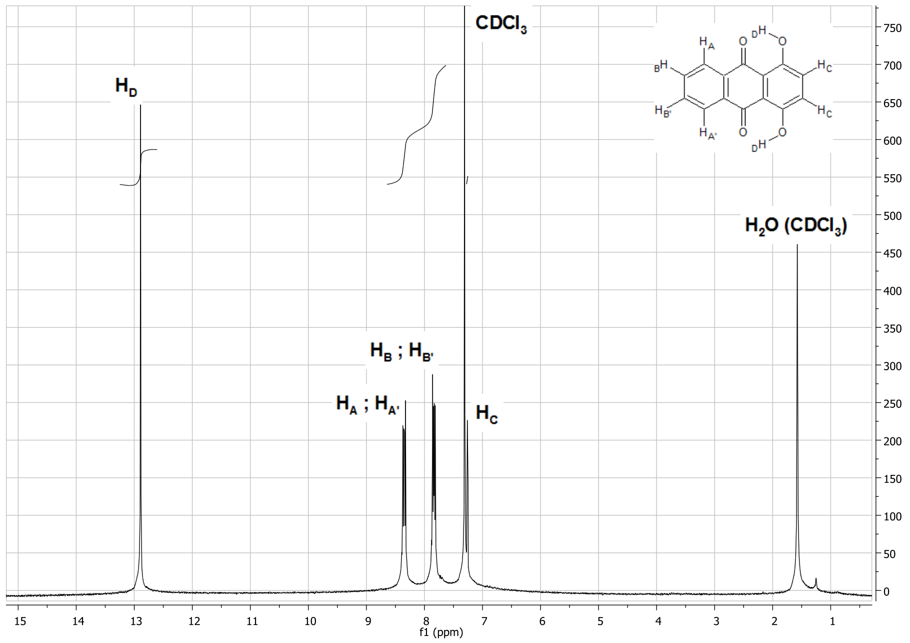

Figure S4. ^{1}H NMR (CD$_6$Cl3) spectrum of Quinizarin (1,4-Dihydroxy-9,10-anthraquinone): 12.9 ppm (s, 2H); 8.3 ppm (dd, *J* = 2Hz , 2H); 7.8 ppm (dd, *J* = 2Hz , 2H); 7.3 ppm (s, 2H).

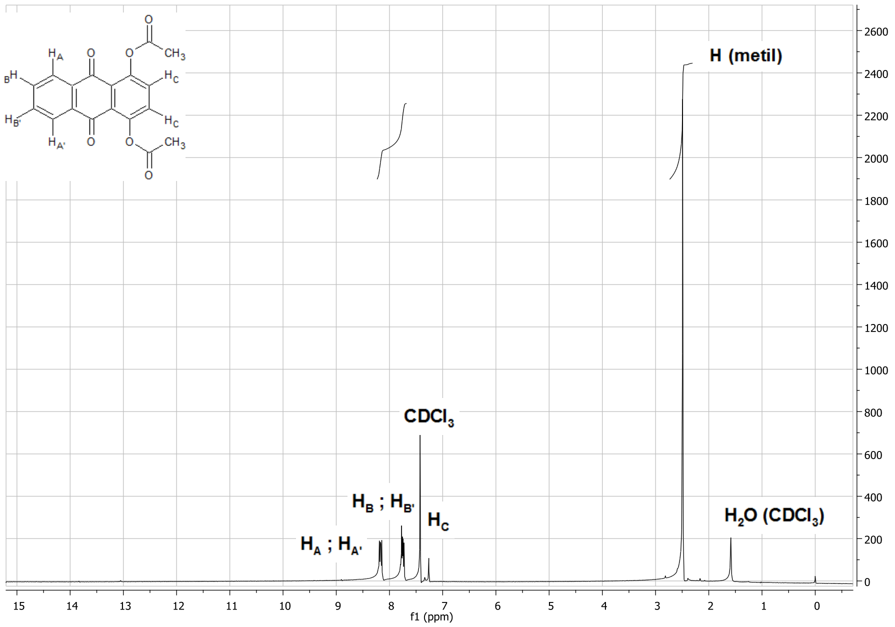

Figure S5. ^{1}H NMR (CD_6Cl_3) spectrum of Quinizarin diacetate (9,10-Anthracenedione,1,4-bis(acetyl oxy)): Aromatics: 8.2 ppm (dd, *J* = 2Hz, 2H); 7.7 ppm (dd, *J* = 2Hz, 2H); 7.3 ppm (s, 2H); Aliphatics: 2.5 ppm (s, 6H).

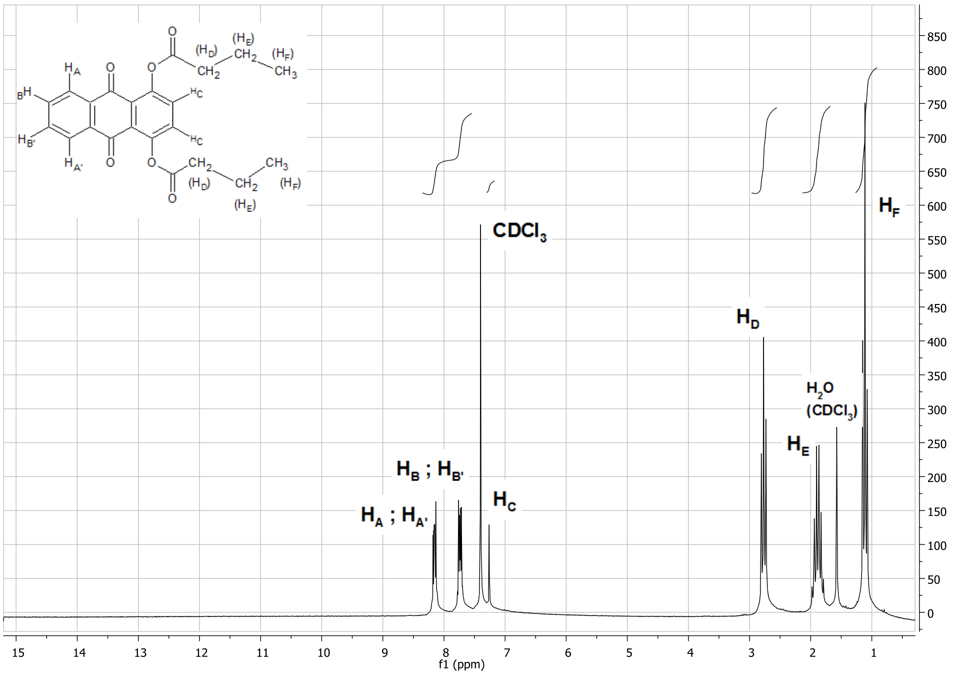

Figure S6. ^{1}H NMR ($CDCl_3$) spectrum of Quinizarin dibutyrate (9,10-Anthracenedione,1,4-bis(butyryl oxy)): Aromatics: 8.2 ppm (dd, *J* = 2Hz, 2H); 7.7 ppm (dd, *J* = 2Hz, 2H); 7.3 ppm (s, 2H); Aliphatics: 2.8ppm (t, *J* = 8Hz, 4H); 1.9 ppm (sext, *J* = 8Hz, 4H); 1.1 ppm (t, *J* = 7Hz, 6H).
